# Internal Revenue Professional Income

**Published:** 1921-02

**Authors:** 


### INTERNAL REVENUE PROFESSIONAL INCOME

### HOW THE FEDERAL INCOME TAX APPLIES TO THE MAN OF THE PROFESSIONS

To the professional man the problem of correctly making out an income tax return for the year 1920 is somewhat more involved than that presented to the salaried man. The wage earner on a fixed salary has an accurate estimate of the amount of compensation received for personal services, while the professional man's income varies from year to year. In the professional class may be included the physician, dentist, lawyer, architect, veterinarian, author and clergyman. Each must figure up his net income for the last year. If single or if married and not living with his wife and his net income was $1,000 or more, or if married and living with his wife and his net income was $2,000 or more, a return must be filed.

The exemptions are the same as for the year 1919, $1,000 for single persons and $2,000 for married persons living with husband or wife, and heads of families, plus $200 for each person dependent upon the taxpayer if such persons are under eighteen years of age, or incapable of self-support because mentally or physically defective. The period for filing returns is from January 1 to March 15, 1921.

The professional man must make a return of all fees, salaries and other compensation for services rendered, together with income from all other sources. If he keeps his accounts on the "receipts and disbursements" basis—which means a record of the amount received and the amount paid for expenses—he should file his income tax return for the year 1920 on that basis. If he keeps books showing income accrued and expenses incurred during the year, he must make his return from his books and include all income, even though not entered on his books. If books are kept on the accrual basis the taxpayer must include all income that accrued, even though not actually received, and may deduct items of expense, although not actually paid. Both the receipts and disbursement basis and the accrual basis are explained in instructions on the forms for filing individual returns of income.

This constitutes gross income from which the taxpayer is allowed certain deductions in arriving at net income upon which the tax is assessed. Among such deductions are the cost of supplies used by him in the practice of his profession, expenses paid in the operation and repair of an automobile used exclusively in making professional calls, dues to professional societies and subscriptions to professional journals, rent paid for office room, expense of fuel, light, water, telephone used in his office, and the hire of office assistants. Amounts expended for books, furniture and professional instruments and equipment of a permanent character are not allowable deductions. In the case of a professional man who maintains an office, but incidentally receives at his home patients, clients, or other callers in connection with his professional work, no part of the rent of the home is deductible. If, however, he uses part of the house for his office such portion of the rent as is properly attributable to such office is a deductible item.

A reasonable allowance is made for depreciation, or wear and tear of equipment and instruments used by professional men. When through some new invention or radical change in methods or similar circumstances, the usefulness in his profession of some or all of his instruments or other equipment is suddenly terminated, so that he discards such asset permanently from use, he may claim as a loss for that year the difference between the cost (reduced by reasonable adjustment for wear and tear it has undergone), and its junk or salvage value. If the apparatus was owned prior to March 1, 1913—the date the first income tax law became effective—its fair market value at that date should be considered instead of its cost in figuring depreciation and obsolescence.

Deductions for uncollectible fees form an important item in the returns of many professional men. To be allowed as a deduction, a debt must be worthless and must have been charged off within the year in which its worthlessness was discovered. The return must show evidence of the manner in which discovery was made. For example, statement should be made that the debtor has been discharged from bankruptcy or has disappeared leaving no trace, or that all ordinary means of collections have been exhausted.

A debt proved to be worthless is not always a proper deduction. Unpaid amounts representing fees for professional services are not allowed as deductions unless included as income in the return for the year in which the deduction is sought or in a previous year. The fact that expected income was not received does not reduce the taxable income. If a debt is forgiven it can not be deducted, because it is then regarded as a gift. A debt may not be charged off or deducted in part, but must be wholly worthless before any part can be deducted.

Compensation in any form for professional services must be included as income. If a physician, lawyer, or other professional man should receive from a merchant, goods in payment for professional services, the fair market value of such goods must be included as net income.

Forms for filing returns are now available at offices of collectors of internal revenue and branch offices. Collectors will mail to each person who last year filed a return a copy of the return form for 1920. Failure to receive a form, however, does not relieve a taxpayer of his obligation to file a return and pay the tax on time. Taxpayers whose net income for the year 1920 was $5,000 or less should use Form 1040A. Those whose net income was in excess of $5,000 should use Form 1040.

In addition to the individual forms, partnerships must file a return of income, or even if there was no net income, on Form 1065. Partnerships as such are not subject to the income tax. Individuals carrying on business in partnership, however, are taxable upon their distributive shares of the net income of such partnerships whether distributed or not and are required to include such shares in their individual returns. The return must show the name and address of each partner and his share of net income.

The tax this year as last may be paid in full at the time of filing the return—on or before March 15, 1921—or in four equal installments, due on or before March 15th, June 15th, September 15th, and December 15th. Payment may be made by cash, money order or check, which should be made payable to "Collector of Internal Revenue." The return must be filed with the collector for the district in which the taxpayer lives or has his principal place of business. Heavy penalties are provided by the revenue act for failure to file a return and pay the tax within the time prescribed by law.

